# Reversibility of Polyester Adsorption on Glass^1^,^2^

**DOI:** 10.6028/jres.067A.059

**Published:** 1963-12-01

**Authors:** Roben R. Stromberg, Warren H. Grant

## Abstract

The adsorption of poly(ethylene *o*-phthalate) from chloroform solution on glass powder and aluminum oxide was studied. The adsorption of a number of fractions, varying in number average molecular weight from 970 to 6250 showed a decrease in the moles of polymer adsorbed with increase in molecular weight. The results are interpreted to indicate that this polymer molecule lies in a relatively flattened conformation on the glass surface. More polymer was adsorbed on glass powder at 50 °C than at 0 °C. Adsorption on glass powder that had been outgassed to remove adsorbed water was less than on untreated glass. Initial adsorption at one temperature followed by exposure at the other temperature resulted in complete reversibility of sorption on the untreated glass. Decreasing the temperature from 50 to 0 °C resulted in desorption from the outgassed glass, but increasing the temperature did not result in additional adsorption. These differences are ascribed in part to adsorption across an adsorbed water layer on the untreated glass. An explanation for the “one-direction reversibility” observed for the outgassed glass is presented.

## 1. Introduction

Most studies of polymer adsorption have been concerned with relatively high molecular weight materials. Their behavior is, of course, very different from that of small molecules. Molecules of intermediate molecular weight are also of interest, but have not been extensively investigated. In previous work in this area, the adsorption of a series of relatively low molecular weight poly (ethylene glycols) on a porous carbon [1] 3 and of several polyesters with molecular weights of the order of 4000 on glass, silica, and alumina [2] has been studied. The adsorption isotherms for the polyesters were not nearly as steep at low concentrations as were those for higher molecular weight polymers; a plateau was not attained until relatively high solution concentrations were reached.

The adsorption-desorption behavior of potymer molecules has been of interest, in part, because of the information implied regarding the conformation of the polymer molecule and interactions with the surface. Polymer molecules have been reported to adsorb relatively rapidly [2, 3, 4, 5] on nonporous surfaces, although some recent measurements indicate that this is not necessarily so under all conditions [4, 6]. A recent theoretical treatment [7, 8] has concluded that times to equilibrium are long, caused in part by rearrangements of the molecule at the surface. The times required for desorption, using the same solvent, can be of a different order of magnitude from those required for adsorption. For some systems no desorption occurs within practical units of time (see, e.g., ref. [2]), while for others some desorption does occur [3, 5, 6]. In one study, Soxldet extraction removed only negligible amounts of polystyrene that had been adsorbed on carbon black [9]. In systems in which no desorption occurred with the same solvent used for adsorption, it has been shown that it is possible to remove the adsorbed polymer by using a better solvent [2, 3].

The effect of temperature on the amount of polymer adsorbed is, in general, not very large; both increases and decreases as a result of increasing the temperature have been reported. It also has been shown that it is possible to desorb some polymer by means of changes in temperature. Poly-(neopentyl succinate) is for all practical purposes “irreversibly” adsorbed when glass is used as the substrate and the same solvent used for desorption [2]. Adsorbing the polyester at one temperature and changing the temperature demonstrated that polymer could be desorbed by a temperature increase, but that decreasing the temperature did not readsorb the polymer [5]. Similar measurements were made with poly(methyl methacrylate) and polyvinyl acetate) adsorbed on iron from several solvents [10]. The behavior of these systems was similar. The “reversibility” of the adsorption was dependent upon the direction from which the temperature change was effected.

It has not been clear whether the polymer displaces a molecule such as water from the surface of a solid before it is adsorbed itself, is adsorbed on top of the water molecule, or perhaps is prevented from being adsorbed by the adsorbed water. In the case of polystyrene adsorbed on carbon, it was found that predrying tends to decrease the amount of polymer adsorbed [9, 11].

This paper reports the results of a study of the molecular weight dependence, the reversibility of adsorption as a function of temperature, and the effect of adsorbed water on the adsorption of poly (ethylene *o*-phthalate) on glass surfaces.

## 2. Experimental Procedure

### 2.1 Materials

The polymer used in this study was the linear saturated polyester, poly (ethylene *o*-phthalate). It was prepared by refluxing ethylene glycol and phthalic anhydride in a molar ratio of 1: 1 for various times to obtain the desired average molecular weights. Four such preparations were made,^4^ covering a range of molecular weights. After purification, each of these preparations was individually fractionated from 0.5 percent solutions of polymer in acetone with water as the nonsolvent.

The number average molecular wieghts ( ${\bar{M}}_{n}$) of the fractions were obtained by end-group analysis. Preparation 1 was fractionated into 10 fractions, from which a fraction with ${\bar{M}}_{n}=970$ was selected. Preparation 3 was fractionated into 9 fractions, from which those with ${\bar{M}}_{n}=2300$, 3100, and 4300 were selected. Portion 4 was fractionated into 8 fractions, two of which with ${\bar{M}}_{n}=5450$ and 6250 were used. After fractionation the polymers were precipitated from chloroform solution with ether and dried in a vacuum oven at 80 °C for approximately 18 hr.

X-ray analysis did not show any evidence of crystallinity. The fraction with the molecular weight of 6250 was used to study the effect of drying and of temperature changes.

Chloroform was the solvent for all adsorption measurements. It was distilled from calcium chloride prior to use. For those experiments in which moisture was to be rigorously excluded, the chloroform was vacuum distilled after drying over calcium hydride.

The principal adsorbent was Pyrex glass power, 325 mesh. The entire quantity of glass powder was mixed to obtain a homogeneous mixture, evacuated for several days in individual tubes, and sealed under vacuum for storage. In this manner the surface of the glass was protected from attack by the atmosphere during storage. Each tube contained approximately enough glass powder to obtain a complete adsorption isotherm. The surface area 5 of the glass as determined by BET nitrogen adsorption was 1.8 m^2^/g. The other adsorbent used was aluminum oxide 6 with a surface area (see note 5) of 55 m^2^/g.

### 2.2 Procedure

The amount of polymer adsorbed was determined by measuring the change in the concentration of the polymer solution, using the carbonyl absorption band at approximately 5.8 *μ*. Measurements were made in a double-beam infrared instrument in a cell approximately 0.4 mm in length.

The experiments in which rigorous drying of the material was not a factor were carried out by pipetting 20 ml of polymer solution that had been maintained at a constant temperature into a tube containing 10 g of glass powder or 0.3 g of Al_2_O_3_ that had also been maintained at the same temperature. After filling, the tubes were maintained at the same temperature and sealed with a gas torch. The sealed tube was then rotated end over end in a constant temperature bath for a length of time that depended on the specific experiment but was at least 20 hr in all cases. The solution and glass powder, therefore, were at the same temperature both before and during the initial portion of an adsorption experiment. After the elapsed adsorption time, the rotation was stopped, the glass allowed to settle at the same constant temperature, and in the experiments in which drying was not involved, the tubes were opened and a portion of the solution removed for analysis. If the adsorption mixture was to be reexamined or retreated, 1 ml of the solution was removed for analysis and the tube resealed. The small amount of glass powder that remained suspended in the solutions to be analyzed was removed by centrifugation.

For those experiments designed to study the effect of temperature change on the amount of polymer adsorbed, tubes were filled as described above and rotated at a constant temperature of 50 ±0.5 °C and 0±0.5 °C for approximately 20 hr. The powder was then allowed to settle at constant temperature, the tube opened, 1 ml of solution removed for analysis, and the tube resealed. The adsorption tubes were then placed in a bath at the other temperature and rotated for approximately 44 hr. The glass was again allowed to settle at the constant temperature, the tube reopened and 1 ml of solution again removed for analysis. In some cases the tubes were resealed and rotated at the original temperature for an additional 44 hr, and the solution analyzed again after this time.

Some of the adsorption runs were carried out on glass powder that had been pretreated to remove water and other gases from the surface. The powder was outgassed under a vacuum of about 10^−5^ mm of Hg at a temperature of 250 °C for 48 hr and polymer solution added without exposing either adsorbent or solution to the atmosphere. This was accomplished by connecting, by means of break-off seals, a tube containing polymer solution to each tube containing glass powder. During the time that the adsorbent was outgassed, the tubes containing the polymer solution were evacuated, the solvent distilled from the polymer, dried over calcium hydride and redistilled back into the calibrated polymer tube. In this manner water was removed from the glass, solvent, and polymer. The assembly was removed from the vacuum system and brought to constant temperature (50 or 0 °C) prior to mixing. The adsorption was carried out as described above. In order to remove solution for analyisis without exposure of the glass-solution system to the atmosphere, small calibrated tubes were preattached to each adsorption tube. It was possible, therefore, after one phase of the adsorption experiment, to pour 1 ml of solution into such a tube for analysis, and seal it from the system. In this manner the remaining solution was not exposed to the atmosphere prior to rotation at another temperature.

## 3. Results

**Figure 1:**
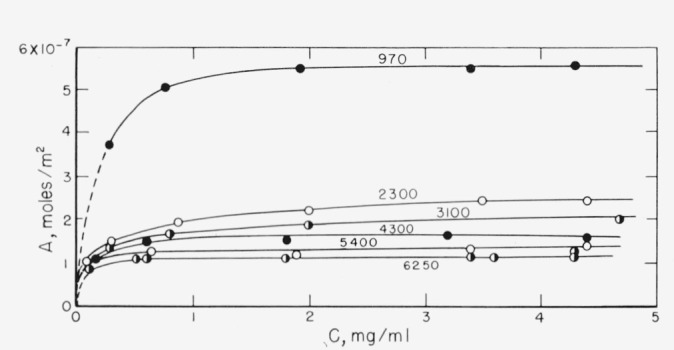
Adsorption isotherms of 6 molecular weight fractions of poly (ethylene o-phthalate) on glass powder. The moles adsorbed per *m*^2^ are plotted against the final solution concentration. The molecular weights given are number average; the adsorption temperature was 50 °C, and the solvent chloroform.

The adsorption isotherms at 50 °C of six fractions of polyethylene *o*-phthalate) in chloroform solution, ranging in molecular weight from 970 to 6250, are given in figure 1. The largest difference among the curves is between that of the low molecular weight of 970, which represents about 5 repeating units, and that of the molecular weight of 2300. The curves for molecular weights ranging form 2300 to 6250 are grouped relatively close together; there is a definite although small dependence on molecular weight for this range. It is realized, of course, that the surface area determined from nitrogen adsorption is probably not the actual surface area available to the polymer. The molecular weight of the polymer fractions used was not very large, and the actual area available, although probably smaller than that determined by nitrogen adsorption, may not be too different. It is also reasonable to expect that the fraction of surface area available does not change significantly for the range of molecular weights studied.

**Figure 2:**
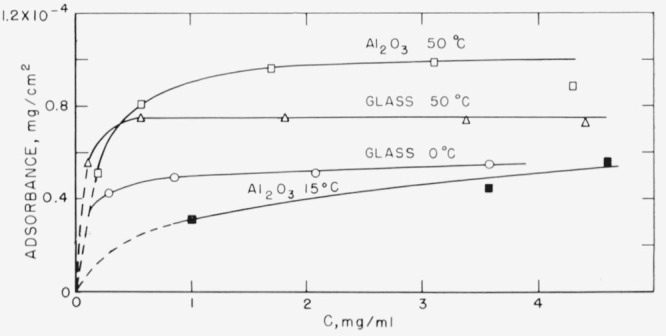
Absorbance of ${\bar{M}}_{n}=6250$ fraction at 0 and 50 °C on glass powder, and at 50 and 15 °C on Al_2_O_3_. The powders were exposed to air prior to adsorption. △ Glass 50 °C ◯ Glass 0°C □ A1_2_O_3_ 50 °C ■ A1_2_O_3_ 15 °C

**Figure 3:**
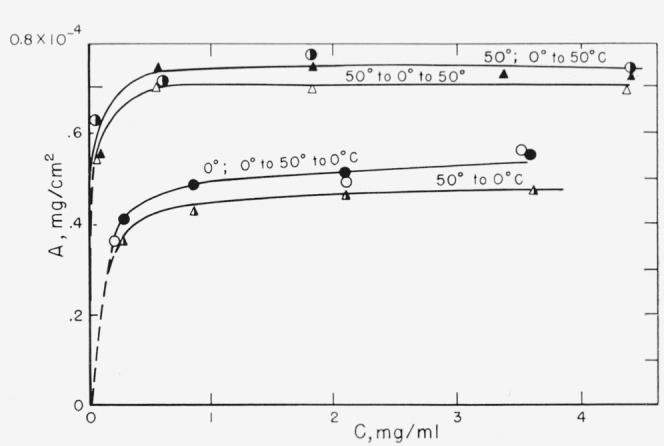
Effect of temperature cycle on adsorption isotherms of ${\bar{M}}_{n}=6250$ fraction from chloroform solution on glass powder. Glass powder exposed to atmosphere prior to contact with polymer solution. ▲ 50 °C ◮50 to 0°C △ 50 to 0 to 50 °C ● 0°C ◑0 to 50 °C ○ 0 to 50 to 0 °C

The adsorption isotherms of polyethylene *o*-phthalate) from chloroform solution on the glass powder at 0 and 50 °C and on Al_2_O_3_ at 50 and 15 °C are shown in figure 2. The adsorbent powders were exposed to air before the adsorption. More polymer is adsorbed as the temperature is increased. When the temperature is changed after adsorption has taken place, as described in the experimental portion, the results shown in figure 3 are obtained. The glass had been exposed to the atmosphere prior to the initial adsorption, and the tubes opened to the atmosphere for sampling prior to being placed in a bath at another temperature. Essentially complete reversibility occurs when the temperature is cycled. When the adsorption is carried out at 50 °C initially and then changed to 0 °C, the isotherm shifts from that of 50 °C, to that of 0 °C. When the temperature is again changed back to 50 °C, the isotherm again shifts back to the original isotherm of 50 °C. The same reversibility occurs when 0 °C is the initial adsorption temperature.

**Figure 4:**
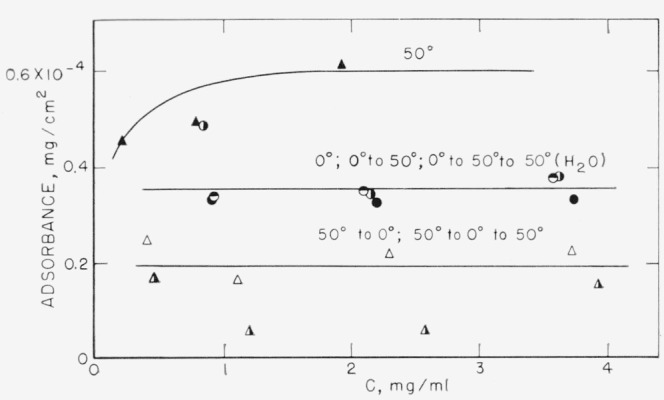
Effect of temperature cycle on adsorption isotherms obtained on glass after pretreating the glass by outgassing and heat. Adsorptions and temperature cycles carried out without exposing the glass powder to the atmosphere. Solvent- chloroform; polymer fraction ${\bar{M}}_{n}=6250$. ▲ 50 °C ◮ 50 to 0 °C △ 50 to 0 to 50 °C ●0°C ◑0 to 50 °C ◓ 0 to 50 to 50 °C in presence of added water

The results of heating and outgassing the glass powder prior to adsorption are shown in figure 4. A decrease in the adsorbance at bot h 50 and 0 °C was observed as a result of the pretreatment of the glass powder. The effect of the temperature cycling was also altered as a result of the heating and outgassing. Changing the temperature from 50 to 0 °C again resulted in a shift in the isotherm to a lower adsorbance. Changing the temperature back to 50 °C, however, did not result in an increase in the amount adsorbed. When the adsorption was initially carried out at 0 °C and the temperature then changed to 50 °C, the isotherm remained at the same location; no additional polymer was adsorbed to increase the adsorbance to that obtained when the adsorption was initially carried out at 50 °C.

In order to determine the effect of water added after adsorption had taken place on a “nonreversible system,” tubes in which adsorption had initially occurred at 0 °C and which had then been changed to 50 °C were opened, 1 ml of water added, and the tube sealed and rotated at 50 °C for an additional 44 hr. The addition of this water did not cause additional polymer to be adsorbed to equal the absorbance of the 50 °C isotherm, as can be seen from figure 4.

## 4. Discussion

### 4.1. Conformation on Adsorbent

**Figure 5:**
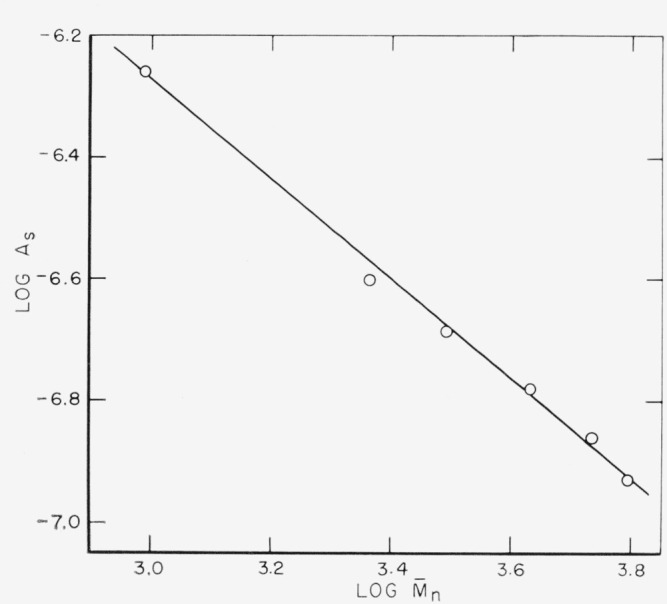
Plot of *log A* versus *log* ${\bar{M}}_{n}$ to determine the effect of molecidar weight on the amount of polymer adsorbed.

The adsorption of polydimethylsiloxanes [12] and poly (methyl methacrylate) [10] has been found to fit the empirical equation *A=KM*^a^ where *A* is the amount of polymer adsorbed at the plateau of the isotherm, *M* is the molecular weight in relative units, and *K* and *a* are constants. If the log of *A* is plotted against the log of *M_n_*, a straight line is obtained for the fractions of poly (ethylene *o*-phthalate), as shown in figure 5. The values of *K* and *a* obtained from a least squares calculation are

$$\begin{array}{c} K=1.38\times 10^{-4}\text{moles}/m^{2},\\ a=-0.81. \end{array}$$

These values can be compared with those obtained using the other polymer systems. The size of *a* is an indication of the conformation of the polymer on the surface. In the case of poly (methyl methacrylate) [10], the values of *a* were very close to zero for two solvents and two adsorbents. For the polydimethylsiloxanes [12], the values of *a* varied from 0.23 to 0.40, depending on the solvent and adsorbent. Both of these sets of values of *a* were obtained for *A* expressed in the *weight of the polymer.* If *A* were expressed in moles per unit area, the values for poly (methyl methacrylate) would become close to −1 and those for the polydimethylsiloxanes would vary from −0.77 to −0.60.

As seen in figure 1, the number of moles of polymer adsorbed decreases with increasing molecular weight, giving rise to *negative* values of *a.* The range of possible values of *a*, when *A* is expressed in *moles per unit area* is −1≤*a*≤0. When *a*=0, the empirical equation shows that the number of molecules adsorbed is independent of molecular weight. This can be interpreted [12] to mean that the polymer molecules would be attached at one location only. When *a*= −1, they would be flat on the surface, assuming “monolayer” adsorption.

The values of *a* for poly(ethylene *o*-phthalate) is between these two limits, but is much closer to the limit −1. The number of moles adsorbed per unit area would be expected, therefore, to be much more sensitive to changes in molecular weight at relatively low molecular weights, and much less sensitive at higher molecular weights. This can be seen from figure 1 to be the case.

These results would indicate, then, that the polyethylene *o*-phthalate) molecule lies in a relatively flattened conformation on the glass surface, but nevertheless still has some extension into the solution.

It appears that the conformation of the molecule at the surface is very dependent on the specific system studied if the value of *a* can be used as a criterion. For poly (methyl methacrylate) [10] the value of *a* was very close to zero, indicating such a flat array for the systems studied. On the other hand, the polydimethylsiloxanes [12] gave values between 0.23 and 0.43.

In table 1 are listed the degree of polymerization for the different molecular weights studied here and the area available per molecule and per segment, i.e., repeating ester unit. At the lower molecular weights, each repeating unit has available to itself an area of approximately 55 to 60 Å^2^. This is only slightly less than the areas determined for a series of polyesters on aqueous surfaces [13, 14]. It would appear then, that at the low molecular weights, the polymer molecule is able to spread on the surface relatively freely, and to lie relatively flat, similar to its conformation on a water surface where a large amount of freedom of motion is possible.

In the case of the larger molecular weights studied here, it appears that a limiting surface area of the order of 45 Å^2^ is available per segment. This area is still relatively large, implying that the molecule lies relatively flat on the surface with only small loops extending into the solution. It should be noted that these conclusions regarding the flatness of the adsorbed molecule are based on a uniform surface available to the molecule. If the entire surface is not available to the molecule for adsorption, then the loops extending into the solution must be larger, of course.

### 4.2 Reversibility of Sorption

It was shown in figure 2 that the adsorbance of poly (ethylene *o*-phthalate) from chloroform solution was greater at higher temperatures than at lower temperatures. It had been reported earlier [2] that less polyester was adsorbed from toluene solution at higher temperatures. It is probable that this observed decrease in the adsorbance from toluene, a poor solvent, was caused by a large increase in the polymer-solvent interaction brought about by the higher temperature. Therefore, what was most probably measured was not simply the effect of two different temperatures, but essentially adsorption from what was almost two different solvents, a poor (low-temperature) and a relatively good (high- temperature) solvent. This effect would be negligible for chloroform. Therefore, the larger adsorption at the higher temperature is probably the normal condition to be expected for polyester adsorption on these oxide surfaces. It is realized, of course, that differences in adsorbent-solvent interaction may also be involved.

Work previously reported has shown that cycling the temperature resulted in adsorption that was dependent upon the temperature path followed. It is realized that given sufficient time, equilibrium conditions would be established and the systems discussed below would all be reversible. The dependence upon path, therefore, refers to the observed results and what appears to be “nonreversible” actually means an extremely slow rate of desorption. For a polyester system in which toluene was the solvent, there was a decrease in adsorbance when the tern- temperature was raised, but the polymer did not readsorb when the temperature was lowered [5]. A similar behavior was observed for poly (vinyl acetate) and poly(methyl methacrylate) [10]. The three systems were similar in that polymer could be desorbed, but additional polymer could not be readsorbed. The system poly (ethylene *o*-phthalate) - chloroform-glass described in this paper exhibited somewhat different behavior. When the glass powder had been exposed to the atmosphere, the system was completely reversible. However, when the glass was pretreated by drying, the system was no longer completely reversible. In this case also polymer was only desorbed, although the desorption was brought about by decreasing, rather than increasing, the temperature.

The surfaces of amorphous silica and silica gel have been studied by means of infrared spectrophotometry, especially in relation to the nature of the adsorbed water and bound water, and the effect of temperature on this water [15, 16, 17]. Water vapor was found to adsorb physically on the silanol sites only and not on the remainder of the surface [15]. Rehydration of the surface was completely reversible up to 400 °C and a change in the nature of the dehydrated surface was indicated above this temperature. It was also reported [17] that “bound water’’ in silica gel consists entirely of SiOH groups located on the surface and that the spectrum of physically adsorbed water resembled that of liquid water. Further, outgassing below 300 °C did not change the number of surface silanol groups [16]. Therefore, our outgassing of glass at 250 °C should have removed only the physically adsorbed water layer and should not have appreciably changed the character of the glass surface.

It would appear, then, that the adsorption of the poly (ethylene *o*-phthalate) onto the untreated glass, and the complete reversibility of adsorbance as a result of temperature cycling, as shown in figure 3, could result from adsorption of the polymer, not onto the glass surface directly, but rather onto the preadsorbed water layer. As mentioned earlier, the glass surface area available per segment is not too different from that expected on a water surface. The polymer could be expected to adsorb and desorb relatively readily from such a “water” surface.

As described above, dehydration should remove the adsorbed water layer, leaving a surface consisting, to a large extent, of silanol groups. Adsorbed water interacts with these surface silanol groups through hydrogen bonds [16]. It is reasonable to suppose that the polyester would also be bound to this dehydrated surface by hydrogen bonds. A different surface, therefore, is available to the polymer after outgassing of the glass powder, and the nature of the adsorptive bond may be somewhat different. As was observed in figures 3 and 4, the outgassing results in a decrease in the adsorbance. This may be a result of fewer adsorption sites available on the glass surface.

In the case of the pretreated glass, it is possible that the total number of sites on the glass occupied by polyester at both temperatures is approximately the same. The differences in the adsorbance are related to the different number of sites occupied per polymer molecule, i.e., the conformation of the polymer molecule. This an oversimplification, of course, and all factors such as differences in solvent-adsorbent interaction, etc., should be considered. When the temperature is lowered, desorption does take place, as required for equilibrium. The remaining polymer molecules rearrange so as to occupy the vacated sites.

When the temperature is again raised, the equilibrium situation calls for a somewhat different polymer conformation. As each polymer molecule now occupies more area than allowed at equilibrium, polymer must be desorbed before additional polymer can be readsorbed. It seems apparent that under these circumstances, the desorption required to achieve the new polymer conformation proceeds at an extremely slow rate, resulting in what appears to be an irreversible condition. Addition of water to the system, figure 4, did not dislodge the adsorbed layer.

It is apparent from the discussion and possibilities presented here that much more knowledge is necessary about the conformation of the polymer molecule. The thickness of adsorbed polymer films *in situ* has been recently investigated using ellipsometry [18]. The system studied was polystyrene of molecular weight 76,000 adsorbed on a chrome-chrome oxide surface from cyclohexane near the theta temperature. For solution concentrations of the order of 1 to 6 mg/ml, it was determined that the polymer was adsorbed as a highly swollen film containing about 12 g/100 ml of polymer and extending about 200 A into the solution. It is apparent that studies of this nature must be extended to the systems described here, in which both the adsorbent surface and the polymer molecule have more nearly the same polar character.

## Figures and Tables

**Table 1:** Glass surface area available for adsorption of poly (ethylene o-phthalate)

${\bar{M}}_{n}$	Degree of polymerization	Adsorption	Area	Area
				
		*Moles/m*^2^	*Å*^2^/*molecule*	*Å*^2^*/segment*
970	5	5.5 × 10^−7^	300	60
2300	12	2.4	690	57
3100	16	2.0	850	54
4300	22	1.7	970	44
5400	28	1.3	1260	45
6250	32	1.2	1430	45

## Footnotes

3 Figures in brackets indicate the literature references at the end of this paper.

4 Polymer prepared by H. L. Steinberg, National Bureau of Standards.

5 The surface areas were determined by Dr. C. M. Hunt and Mr. A. W. Woolf, National Bureau of Standards.

6 Alon C, provided by the Godfrey L. Cabot Corp.
